# Coping with bowel dysfunction after low anterior resection for rectal cancer: A qualitative synthesis

**DOI:** 10.1016/j.apjon.2025.100787

**Published:** 2025-09-18

**Authors:** Eun Young Kim, Min Jeong Kim, Young Man Kim

**Affiliations:** a Department of Nursing, Soonchunhyang University, Cheonan-si, Republic of Korea; b Department of Nursing, Graduate School, Jeonbuk National University, Jeonju-si, Republic of Korea; c Red Cross College of Nursing, Chung-Ang University, Seoul, Republic of Korea

**Keywords:** Colorectal surgery, Defecation, Meta-analysis, Qualitative research, Rectal neoplasms, Systematic review

## Abstract

**Objective:**

This study synthesized existing qualitative research on patients’ experiences of bowel dysfunction following low anterior resection for rectal cancer and examined the strategies they use to cope and adapt to related challenges.

**Methods:**

A comprehensive search was conducted across PubMed, EMBASE, CINAHL, SCOPUS, Web of Science, and the Cochrane Library for qualitative studies published up to June 2025, supplemented by reference screening. A meta-ethnographic approach was employed to integrate findings interpretively while preserving the contextual depth of individual studies.

**Results:**

Twenty-two studies involving 415 participants were included. Three overarching themes emerged: (1) living with uncertainty, (2) experiencing social isolation and disconnection, and (3) striving to establish a new balance in life. Patients reported unpredictable bowel symptoms that disrupted daily activities, generating uncertainty, social withdrawal, and psychological distress. Despite these challenges, patients gradually adapted through self-care practices, emotional support, and psychological acceptance, which facilitated a shift toward stability and improved quality of life.

**Conclusions:**

Bowel dysfunction after low anterior resection imposes substantial physical, psychological, and social challenges on patients. Supporting adaptation requires patient-centered nursing interventions that extend beyond symptom management to provide comprehensive psychosocial support, promote self-care, and enhance long-term well-being.

**Systematic review registration:**

PROSPERO CRD42024590342.

## Introduction

Colorectal cancer is the third most common cancer worldwide, with approximately 1.9 million cases reported in 2020, and rectal cancer accounting for 38% of these cases.^1^ Low anterior resection (LAR) is widely performed as a sphincter-preserving surgery that balances oncologic control with functional preservation.^2^ However, LAR frequently results in postoperative bowel dysfunction, collectively termed low anterior resection syndrome (LARS), which includes urgency, incontinence, frequent defecation, and difficulty in stool control.^3^ The prevalence of LARS varies, with major LARS affecting up to 41% of patients.^4^ These symptoms often persist for years and significantly impair the quality of life (QoL).^5^

Despite its profound impact, LARS remains under-recognized and inadequately managed in clinical practice.^4^ Many patients experience unpredictable symptoms without sufficient medical guidance, leading to frustration, social withdrawal, and emotional distress. Although coping strategies, such as dietary modifications and pelvic floor exercises have been suggested, there are no standardized guidelines for managing LARS, leaving many patients without effective solutions.^6^

Existing research on LARS primarily describes common symptoms and coping mechanisms but lacks a deeper exploration of the long-term psychosocial impact and adaptation process. Additionally, prior systematic reviews^7^^,^^8^ have limitations, such as restricted database searches, methodological inconsistencies, and insufficient coverage of recent literature. For example, Pape et al. (2021) limited his systematic review to three databases, potentially overlooking pertinent studies indexed in other databases. Similarly, Yanting et al. (2023) synthesized nine qualitative studies in his systematic review; however, the methodological differences among these studies may have affected the consistency and depth of the analysis. Furthermore, both reviews included only one study published after 2020, which limits their ability to capture evolving patient experiences and recent developments in clinical practice.

Meta-ethnography is an interpretive approach that synthesizes results from diverse qualitative studies to produce unique conceptual frameworks rather than merely aggregating data.^9^ Unlike traditional narrative reviews, meta-ethnography allows researchers to systematically analyze the ways in which different studies construct meanings around complex experiences—in this case, patients’ experience of bowel dysfunction following LAR for rectal cancer. Through the translation and integration of concepts across studies into overarching themes, this method promotes a deeper understanding of adaptation among patients situated in various cultural and clinical contexts. Therefore, the present study utilized meta-ethnography to reflect the nuanced and lived experiences of patients and develop an integrated explanatory model of adaptation.

Patients often experience long-term challenges that require effective self-management of symptoms due to the enduring nature of bowel dysfunction after surgery.^10^ As frontline health care workers, nurses are ideally situated to support patients in managing LARS through conservative approaches such as bowel training, dietary guidance, and emotional support.^10^ Their role is particularly significant in survivorship care, where ongoing, individualized interventions can promote adaptation and enhance patient's QoL.

Therefore, the present study aimed to synthesize the existing qualitative studies to provide a comprehensive understanding of the experiences and adaptation processes of patients living with bowel dysfunction after LAR for rectal cancer and contribute to the development of patient-centered nursing interventions and survivorship care strategies.

## Methods

This study employed a systematic review and qualitative meta-synthesis approach. The study was conducted using the seven-step meta-ethnography approach developed by Noblit and Hare^9^ after protocol registration in PROSPERO (CRD42024590342). The review is reported in accordance with the guidelines for enhancing transparency in reporting the synthesis of qualitative research (ENTREQ).^11^

### Inclusion and exclusion criteria

Studies were eligible for inclusion if they involved adult patients (≥ 18 years) who had undergone LAR for rectal cancer and focused on their experiences of bowel dysfunction, including symptoms, coping strategies, and adaptation processes. Only qualitative studies employing methodologies such as phenomenology, grounded theory, ethnography, content analysis, or narrative inquiry were considered. Included studies were required to explore patients’ perspectives, as well as the psychological and social impacts and challenges associated with bowel dysfunction after surgery. Studies were excluded if they did not involve patients who underwent LAR (e.g., stoma-only populations), did not address bowel dysfunction experiences, or did not use a qualitative design (e.g., quantitative studies, mixed-method studies without extractable qualitative data, reviews, editorials, or commentaries). Studies not published as peer-reviewed original articles or for which the full text could not be obtained despite institutional or author contact were also excluded.

### Search strategy

Six databases were searched: PubMed, EMBASE, CINAHL, SCOPUS, Web of Science, and the Cochrane Library. Additional studies were identified through a manual search and reference screening. Two independent researchers (YMK and MJK) conducted the study selection to ensure methodological rigor. Discrepancies during the screening and selection processes were resolved through discussions and consensus between the researchers.

Search terms included “Colorectal Neoplasms,” “Colorectal Surgery,” “Low Anterior Resection Syndrome,” “Defecation,” “Fecal Incontinence,” and “Qualitative Research,” which were combined and adapted to each database using indexed terms and keywords. A comprehensive search was ensured by employing a non-restrictive approach, including the absence of language restrictions. The final search was concluded on June 2, 2025. To enhance the search validity, a university librarian was consulted during the literature search process. A detailed search strategy is presented in Supplementary Table S1.

### Search outcome

**Fig. 1:**
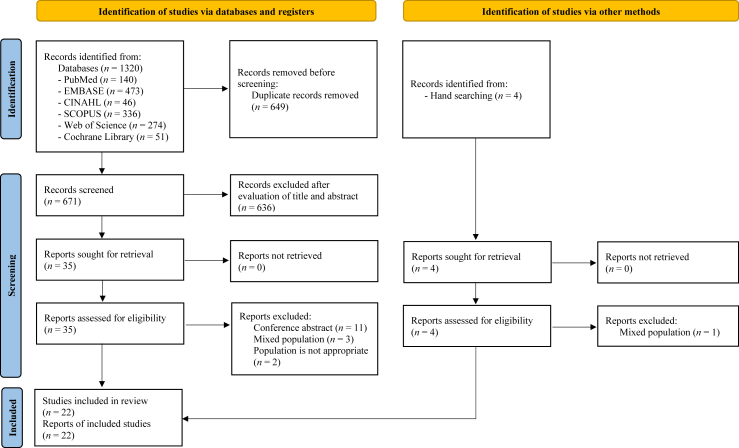
PRISMA flow diagram. PRISMA, Preferred Reporting Items for Systematic reviews and Meta-Analyses.

Database searches identified 1320 studies while manual searches revealed four additional studies. After removing 649 duplicates, 636 studies that did not meet the inclusion criteria were excluded after title and abstract screening. The remaining 35 studies underwent a full-text review. Consequently, 16 studies were excluded for reasons such as abstract-only publications, mixed populations, or ineligible participants. Among the studies identified through the manual search, one was excluded because of mixed population. The final analysis included 22 studies that met the inclusion criteria. Among these, several articles were derived from the same research project, including two publications by Liu et al.^12^^,^^13^ and Pape et al.^4^^,^^14^ and three by Maalouf et al.^15^, ^16^, ^17^ Because these studies reported distinct outcomes and explored different thematic areas, they were treated separately in this review. Participant duplication was avoided by treating the sample characteristics as a single dataset for each research project. When sample characteristics, such as LARS scores or treatment approaches, were reported differently across studies derived from the same research project, data from the original or most detailed report were prioritized. If a sample characteristic was reported in only one study, data available from that study were utilized. The detailed search process and reasons for exclusion are shown in Fig. 1.

### Quality appraisal

The quality of the included studies was assessed using the Critical Appraisal Skills Programme (CASP) checklist.^18^ The CASP checklist comprises 10 items categorized into three sections: (A) validity of the study results, (B) key findings, and (C) applicability of the results to local contexts. The quality of the 22 included studies was assessed by two independent researchers (YMK and MJK). Any discrepancies in assessments were resolved through discussion and consensus.

### Data extraction

The selected studies were arranged in chronological order, and two authors (EYK and YMK) thoroughly reviewed them to gain a comprehensive understanding of the studies. Data relevant to the experience of bowel dysfunction after LAR were extracted, including author details, study objectives, participant characteristics, data collection methods, analysis methods, funding sources, and citations. The extracted data were organized into a user-defined form using Microsoft Excel. To maintain the integrity of the original content, the data were extracted verbatim whenever possible. During the data extraction process, the purpose of the study was kept in mind, and the extraction process was repeated while frequently cross-referencing the original text.^19^

### Data synthesis

The specific data analysis and synthesis followed a meta-ethnographic procedure.^9^ Meta-ethnographic data are composed of three categories: first-, second-, and third-order constructs^20^^,^^21^ First-order constructs refer to the everyday language of the participants in the original study, while second-order constructs refer to the interpretation of the original researcher. Third-order constructs refer to the result of the current researcher's new interpretation by analyzing the first- and second-order constructs of the original study.

In this study, the authors identified and extracted meaningful concepts from the first- and second-order constructs, listed them, and conducted a comparative analysis of the extracted concepts. Concepts with similar meanings were grouped, and the key concepts representing each group were subsequently derived. These key concepts were developed based on the first- and second-order constructs. The derived key concepts were further grouped to generate subthemes, which were then categorized and expressed as third-order constructs, represented as synthesized themes. A reciprocal translation strategy was employed when studies shared similar conceptual structures, while a refutational approach was utilized for contrasting interpretations. During the entire synthesis process, we maintained methodological sensitivity to ensure the preservation of the contextual integrity of each study's findings while refraining from inappropriate generalization across epistemologically distinct frameworks. The two authors (EYK and YMK) independently performed a series of analysis and synthesis processes, compared and analyzed the results at the end of each stage, and reached agreement through discussion in case of differences. Relevant quotations expressing each subtheme were extracted and are presented in the Results section.

## Results

### Characteristics of included studies

As shown in Table 1, 22 studies published between 2006 and 2024 that were selected for this analysis. These studies involved 415 participants, of whom 61% (*n* ​= ​254) were male. Participants’ ages ranged from 28 to 87 years. The studies were conducted in various countries, including the United Kingdom (*n* ​= ​4), Ireland (*n* ​= ​1), Taiwan (*n* ​= ​2), Sweden (*n* ​= ​2), the Netherlands (*n* ​= ​1), China (*n* ​= ​1), Switzerland (*n* ​= ​1), Denmark (*n* ​= ​1), Japan (*n* ​= ​1), Belgium (*n* ​= ​1), Norway (*n* ​= ​1), Canada (*n* ​= ​1), and Spain (*n* ​= ​1).

**Table 1:** Summary of the included studies.

Author, year / Country	Aims	Sample size (Male, %), age (year)	Type of surgery	LARS Score	Treatment approach	Time since surgery	Data collection	Data analysis	Sources of funding
**Desnoo & faithfull, 2006**^23^**/ UK**	To explore how individuals recovered and adapted following surgical resection of their rectal cancer and the syndrome that occurs as a consequence of this operation	7 (28.57) 60 to 78 mean: 70.71	Stoma reversal after anterior resection and loop ileostomy	–	Adjuvant treatment: Chemotherapy (*n* = 4), chemoradiotherapy (*n* = 1)	7–20 months	Semi-structured interview	Constant comparative method	Not reported
**Landers et al., 2012**^24^**/ Ireland**	To explore participants' qualitative perspectives on bowel symptom experiences and management strategies following sphincter saving surgery for rectal cancer	123 (62.60) from 30	Sphincter saving surgery	–	–	6 weeks to 42 months	Semi-structured interview	Deductive Content analysis	Reported (Health Research board Dublin)
**Taylor & bradshaw, 2013**^29^**/ UK**	To explore the lived experiences of patients with anterior resection syndrome, defined as altered bowel function as a consequence of rectal cancer treatment	8 (62.50) 61 to 76 mean: 66.50	TME (*n* = 7), LAR (*n* = 1)	–	–	4–6 weeks	Semi-structured interview	Framework analysis	Reported (National cancer Survivor-ship initiative)
**Lu et al., 2017**^26^**/ Taiwan**	To explore the lived experiences of post-operative patients with rectal cancer experiencing altered bowel function	16 (50) 40 to 75 mean: 55.13	LAR (*n* = 4), stoma closure (*n* = 11), RP (*n* = 1) (Re-stoma, *n* = 1)	–	–	1 week to 36 months	Semi-structured interview	Colaizzi's method	No funding
**Reinwalds et al., 2017**^27^**/ Sweden**	To describe the patient experience during the first 4–6 weeks after reversal of a temporary loop ileostomy owing to RC	16 (43.75) 33 to 81 mean: 63.50	Anterior resection	–	Adjuvant treatment: Chemotherapy (*n* = 8)	4–6 weeks	Semi-structured interview	Thematic content analysis	Not reported
**McCutchan et al., 2018**^32^**/ UK**	To assess the acceptability of rectal irrigation in patients who accept or decline treatment, including a qualitative exploration of the factors influencing their decision, the impact of treatment in terms of QoL, fecal incontinence, and the patient-reported experience	21 (85.71) 36 to 79 mean: 63.57	Anterior resection	Baseline, mean (range) -Treatment group: 35.93 (21–42) -Comparator group: 34.17 (32–37) Six-month follow-up, mean (range) -Treatment group: 17.73 (0–41) -Comparator group: 32.35 (26–37)	Treatment group: rectal irrigation (*n* = 15)	More than 12 months	Semi-structured interview	Thematic analysis	Reported (Innova-tion grant from tenovus cancer care)
**Reinwalds et al., 2018**^28^**/ Sweden**	To illuminate what it means to live with a resected rectum due to rectal cancer, after reversal of a temporary loop ileostomy	10 (40) 56 to 84 mean: 71.60	Anterior resection	–	Adjuvant treatment: Chemotherapy (*n* = 4)	12–20 months	Semi-structured interview	Phenomeno -logical hermeneut-tical method	Not reported
**Van der Heijden et al., 2018**^33^**/ Netherlands**	To explore the impact of LARS from a patient perspective facilitating the construction of a set of recommendations improving current care stratagems	16 (50) 60 to 87	LAR (*n* = 14), Distal sigmoid resection (*n* = 2)	4 weeks after surgery, mean (SD): 21.8 (11.8) Major = 3 Minor = 2 No = 4 Missing data = 7 Time of focus groups, mean (SD): 23.56 (12.9) Major = 6 Minor = 4 No = 4 Missing data = 2	Adjuvant treatment: Chemotherapy (*n* = 2)	Median: 12 months	Semi-structured interview	Inductive content analysis	Not reported
**Liu et al., 2021**^13^**/ China**	To explore the experience of dealing with defecation dysfunction by changing the eating behaviors of people with rectal cancer following sphincter-saving surgery.	36 (63.90)	Sphincter-saving surgery	–	Radiotherapy (*n* = 17)	Up to 24 months	Semi-structured interview	Thematic analysis	Reported (Fudan fuxin founda-tion)
**Buergi, 2022**^22^**/ Switzerland**	To explore challenges of individuals living with LARS 1 year or more after ostomy reversal and formulate a greater understanding of their needs	7 (71.43) 61 to 71 mean:66	Ostomy reversal after LAR	Major = 5, Minor = 2	–	Time since rectum Resection: 15–48 months Time since stoma Reversal: 12–42 months	Semi-structured interview	Interpre-tative phenomeno -logical analysis	Not reported
**Liu & Xia, 2022**^12^**/ China**	To explore the experience of controlling defecation dysfunction among patients with rectal cancer and to understand how patients cope with these symptoms	36 (66.70)	Sphincter-saving surgery	–	–	Up to 24 months	Semi-structured interview	Thematic analysis	Reported (Fuxin fundation of fudan University)
**Laursen et al., 2022**^6^**/ Denmark**	To identify patients' considerations and coping strategies explaining why the burden of major LARS had potentially little or none influence on their QoL	21 (47.62) 50 to 84	LAR	31 to 39	–	3–24 months	Semi-structured interview	Thematic analysis	Reported (Danish cancer Society)
**Nakagawa et al., 2022**^34^**/ Japan**	To investigate the prevalence of defecation dysfunction and the adoption of exercise habits among survivors of rectal cancer, including those with and without stomas	6 (83.30) mean: 75.80	LAR	–	–	Mean ± SD (years): 2.5 ± 1.1	Semi-structured interview	Content analysis	Reported (Japan Society for the promotion of Science KAK-ENHI)
**Pape et al., 2022**^4^**/ Belgium**	To explore the experiences of patients with major LARS with a specific focus on hope and loneliness	28 (64.29) 28 to 77 mean: 54	–	≥ 30	Adjuvant treatment (*n* = 1), Anal irrigation (*n* = 7)	–	Semi-structured interview	Constant comparative method	Reported (Kom op tegen Kanker)
**Tsui & Huang, 2022**^28^**/ Taiwan**	To explore the experiences of losing bowel control in patients who have undergone LAR with sphincter-saving surgery for rectal cancer in taiwan	12 (67) 50 to 67	LAR with sphincter-saving surgery	–	–	Up to 12 months	Semi-structured interview	Colaizzi's method	No funding
**Burch et al., 2023**^2^**/ UK**	To determine the views of people on their health care needs when managing their bowel symptoms following an anterior resection	23 (43.48) 38 to 75	LAR	Median: 37 (13–42)	–	Up to 11 years	Semi-structured interview	Framework analysis	Not reported
**Pape et al., 2023**^14^**/ Belgium**	To explore the information and counselling needs of rectal cancer survivors confronted with major LARS	28 (64.29) 28 to 77 mean: 54	–	≥ 30	–	–	Semi-structured interview	Constant comparative method	Reported (Kom op Tegen Kanker)
**Lovall et al., 2024**^25^**/ Norway**	To shed light on what it means to live with LARS in the first three to six months after colorectal cancer sphincter-preserving surgery	5 (40) 56 to 81 mean: 67.40	PME (*n* = 3), TME (*n* = 2)	Mean: 37.6 (32–41)	Adjuvant chemotherapy (*n* = 1)	Up to 6 months	Semi-structured interview	Giorgi's phenomeno -logical method	No funding
**Maalouf et al., 2023**^15^**/ Canada**	To identify quality-of-life domains most affected by rectal cancer surgery	54 (76) mean: 63.70	LAR (*n* = 41), TATME (*n* = 9), TAMIS/TEM (*n* = 4)	No = 21 Minor = 12 Major = 21	–	Mean (SD), months: 49.5 (32.6)	Semi-structured interview	Content analysis	Reported (Johnson & Johnson, theator, Merck, takeda)
**Maalouf et al., 2024a**^16^**/ Canada**	To determine the content validity of health-related quality-of-life measurement tools in rectal cancer	54 (75.9) mean: 63.70	LAR (*n* = 41), TATME (*n* = 9), TAMIS/TEM (*n* = 4)	No = 21 Minor = 12 Major = 21	–	–	Semi-structured interview	Deductive content analysis	Reported (American Society of Colon and Rectal Surgeons Career development Award)
**Maalouf et al., 2024b**^17^**/ Canada**	To evaluate patients' perspectives on adapting to bowel dysfunction after rectal cancer surgery	54 (76) mean: 63.70	LAR (*n* = 41), TATME (*n* = 9), TAMIS/TEM (*n* = 4)	No = 21 Minor = 12 Major = 21	–	Mean (SD), months: 49.5 (32.6)	Semi-structured interview	Thematic analysis	Reported (Johnson & Johnson, theator, Merck, takeda)
**Ribas et al., 2024**^31^**/ Spain**	To address the educational gaps and support needs of patients with LARS following rectal cancer surgery	6 (50) mean: 54	Rectal cancer surgery with stoma reversal	Major = 6	–	2–12 years	Focus group interview	Thematic analysis	No funding

LAR, low anterior resection; LARS, low anterior resection syndrome; PME, partial mesorectal excision; RP, radical proctectomy; SD, standard deviation; TAMIS, transanal minimally invasive surgery; TATME, transanal total mesorectal excision; TEM, transanal endoscopic microsurgery; TME, total mesorectal excision of the rectum.

The included studies all focused on patients who underwent LAR for rectal cancer, with some specifying whether total mesorectal excision (TME) or partial mesorectal excision (PME) was performed. Nine studies reported LARS scores ranging from 13 to 42. Treatments related to rectal cancer management were described in eight studies, including neoadjuvant and adjuvant therapies such as chemotherapy, chemoradiotherapy, rectal (anal) irrigation, and radiotherapy. Data were collected using semi-structured interviews in 17 studies and focus group interviews in 1 study. Five main analytical techniques were employed across the studies: phenomenological analysis (*n* ​= ​5), thematic analysis (*n* ​= ​6), content analysis (*n* ​= ​6), comparative analysis (*n* ​= ​3), and framework analysis (*n* ​= ​2). Funding sources were reported in eight studies, not reported in six, and explicitly stated as “no funding” in four.

### Quality of included studies

The results of the quality assessment of the reviewed studies are presented in Table 2. Of the 22 studies, 80%, 90%, and 100% of the CASP checklist was met by 4, 9, and 9 studies, respectively. Regarding specific criteria, two studies did not report whether the research design was appropriate to achieve the research objectives, 10 studies did not address whether the relationship between the researcher and participants was appropriately evaluated, one study did not report on ethical issues, and four studies did not specify whether the data analysis was sufficiently rigorous.

**Table 2:** CASP qualitative studies checklist.

Author (year)	Q1	Q2	Q3	Q4	Q5	Q6	Q7	Q8	Q9	Q10	Total score (Y, %)
Desnoo & faithfull (2006)^23^	Y	Y	Y	Y	Y	C	Y	C	Y	Y	**80**
Landers et al. (2012)^24^	Y	Y	Y	Y	Y	C	Y	Y	Y	Y	**90**
Taylor & bradshaw (2013)^29^	Y	Y	Y	Y	Y	Y	Y	C	Y	Y	**90**
Lu et al. (2017)^26^	Y	Y	Y	Y	Y	Y	Y	Y	Y	Y	**100**
Reinwalds et al. (2017)^27^	Y	Y	Y	Y	Y	C	Y	Y	Y	Y	**90**
McCutchan et al. (2018)^32^	Y	Y	C	Y	Y	Y	Y	C	Y	Y	**80**
Reinwalds et al. (2018)^28^	Y	Y	Y	Y	Y	Y	Y	Y	Y	Y	**100**
Van der Heijden et al. (2018)^33^	Y	Y	Y	Y	Y	Y	C	Y	Y	Y	**90**
Liu et al. (2021)^13^	Y	Y	Y	Y	Y	Y	Y	Y	Y	Y	**100**
Buergi (2022)^22^	Y	Y	Y	Y	Y	C	Y	Y	Y	Y	**90**
Liu & Xia (2022)^12^	Y	Y	Y	Y	Y	Y	Y	Y	Y	Y	**100**
Laursen et al. (2022)^6^	Y	Y	Y	Y	Y	Y	Y	Y	Y	Y	**100**
Nakagawa et al. (2022)^34^	Y	Y	Y	Y	Y	C	Y	C	Y	Y	**80**
Pape et al. (2022)^4^	Y	Y	Y	Y	Y	Y	Y	Y	Y	Y	**100**
Tsui & Huang (2022)^28^	Y	Y	Y	Y	Y	C	Y	Y	Y	Y	**90**
Burch (2023)^2^	Y	Y	C	Y	Y	C	Y	Y	Y	Y	**80**
Pape et al. (2023)^14^	Y	Y	Y	Y	Y	Y	Y	Y	Y	Y	**100**
Lovall et al. (2024)^25^	Y	Y	Y	Y	Y	Y	Y	Y	Y	Y	**100**
Maalouf et al. (2023)^15^	Y	Y	Y	Y	Y	C	Y	Y	Y	Y	**90**
Maalouf et al. (2024a)^16^	Y	Y	Y	Y	Y	C	Y	Y	Y	Y	**90**
Maalouf et al. (2024b)^17^	Y	Y	Y	Y	Y	C	Y	Y	Y	Y	**90**
Ribas et al. (2024)^31^	Y	Y	Y	Y	Y	Y	Y	Y	Y	Y	**100**

CASP, Critical Appraisal Skills Programme.

### Synthesized results

The results indicated that patients with rectal cancer who underwent LAR experienced significant difficulties in coping with the uncertainty caused by uncontrollable intestinal problems. These persistent issues often resulted in social isolation and disconnection. Nevertheless, they discovered ways to achieve balance in their lives by actively making an effort to live meaningfully despite ongoing uncertainty.

The three synthesized themes were as follows: a life burdened by uncertainty, living a life of isolation and disconnection, and finding a new balance in life. The key concepts from the first- and second-order constructs, subthemes, and synthesized themes are presented in Table 3.

**Table 3:** Synthesized themes of experiences of bowel dysfunction after LAR among patients with rectal cancer.

Key concepts from the first- and second-order constructs	Subthemes	Synthesized themes
Awareness of changes in physical functions^2^^,^^6^^,^^12^^,^^13^^,^^15^^,^^22^, ^23^, ^24^, ^25^, ^26^, ^27^, ^28^, ^29^, ^30^, ^31^	Challenges posed by unpredictable bodily functions	A life burdened by uncertainty
Loss of control over bowel movement^2^^,^^12^^,^^15^^,^^22^, ^23^, ^24^, ^25^, ^26^, ^27^, ^28^, ^29^, ^30^, ^31^		
A disrupted routine owing to unpredictable bowel patterns^2^^,^^4^^,^^12^^,^^13^^,^^15^^,^^17^^,^^22^, ^23^, ^24^, ^25^, ^26^, ^27^, ^28^, ^29^^,^^31^		
Fatigue from coping with unpredictable symptoms^6^^,^^12^^,^^15^^,^^16^^,^^23^^,^^24^^,^^26^, ^27^, ^28^, ^29^, ^30^		
Activity range limited by restroom availability^4^^,^^6^^,^^10^^,^^12^^,^^15^^,^^17^^,^^22^^,^^26^, ^27^, ^28^^,^^30^, ^31^, ^32^, ^33^	Bowel movements at the core of daily life	
Food choices for bowel regulation^2^^,^^12^^,^^13^^,^^15^^,^^17^^,^^22^^,^^27^^,^^29^, ^30^, ^31^, ^32^		
Family life tailored to accommodate my bowel needs^4^^,^^12^^,^^15^^,^^17^		
Unable to go out owing to irregular bowel issues^4^^,^^12^^,^^15^, ^16^, ^17^^,^^22^^,^^23^^,^^25^^,^^27^^,^^30^, ^31^, ^32^, ^33^	Social isolation	Living a life of isolation and disconnection
Restrictions to hobbies and leisure activities^4^^,^^11^^,^^13^^,^^19^^,^^21^^,^^25^^,^^28^^,^^30^		
Challenges in maintaining intimate relationships^4^^,^^12^^,^^15^^,^^23^^,^^25^^,^^30^^,^^31^^,^^33^		
Reduced social activities owing to social stigma^4^^,^^12^^,^^14^^,^^17^^,^^23^		
Challenges in work life^15^, ^16^, ^17^^,^^25^		
Frustration owing to bowel issues^2^^,^^4^^,^^11^, ^12^, ^13^^,^^18^^,^^24^, ^25^, ^26^, ^27^, ^28^^,^^31^	Psychological distress from emotional instability	
Fear of cancer recurrence^2^^,^^4^^,^^22^^,^^27^^,^^28^		
Feelings of guilt caused by difficulties in sexual relationships^2^^,^^4^^,^^15^^,^^16^^,^^25^^,^^31^		
Fear of my issues being revealed to others^14^^,^^15^^,^^23^^,^^27^		
Confusion from conflicting medical advice^2^^,^^12^, ^13^, ^14^^,^^25^^,^^27^, ^28^, ^29^^,^^33^		
Emotional support from meaningful relationships^2^^,^^6^^,^^14^^,^^17^^,^^23^^,^^25^, ^26^, ^27^, ^28^^,^^31^, ^32^, ^33^, ^34^	Psychological acceptance	Finding a new balance in life
Accepting life as it is^4^^,^^14^^,^^17^^,^^22^^,^^29^		
Feeling grateful for life^6^^,^^15^^,^^17^^,^^23^^,^^25^, ^26^, ^27^^,^^29^		
Finding comfort by comparing with others' suffering^6^^,^^25^		
Become more sensitive to body signals^6^^,^^25^, ^26^, ^27^^,^^31^	Developing personal strategies to regain control	
Accurate symptom management information^2^^,^^14^^,^^15^^,^^17^^,^^25^, ^26^, ^27^^,^^31^^,^^33^		
Dietary management for symptom reduction^2^^,^^6^^,^^13^^,^^15^^,^^17^^,^^22^^,^^23^^,^^25^, ^26^, ^27^, ^28^, ^29^, ^30^^,^^32^^,^^34^		
Use of medication to control symptoms^2^^,^^6^^,^^15^^,^^23^^,^^25^^,^^27^, ^28^, ^29^, ^30^^,^^32^		
Use protective pads to prevent incontinence^2^^,^^17^^,^^22^^,^^23^^,^^25^^,^^28^^,^^29^^,^^32^		
Establishing new bowel habits^6^^,^^15^^,^^17^^,^^22^^,^^25^, ^26^, ^27^, ^28^, ^29^, ^30^^,^^32^	Adapting to a changed daily life	
Building a trusting relationship with health care providers^2^^,^^14^^,^^16^^,^^17^^,^^25^^,^^26^^,^^31^, ^32^, ^33^		
Open communication about illness^2^^,^^6^^,^^14^^,^^16^^,^^17^^,^^25^^,^^28^		
Going out under meticulous planning^6^^,^^13^^,^^15^^,^^17^^,^^22^^,^^32^		

LAR, Low anterior resection.

#### Theme Ⅰ. A life burdened by uncertainty

This theme consists of two subthemes: challenges posed by unpredictable bodily functions and bowel movements at the core of daily life. Patients with rectal cancer experience a life of uncertainty driven by unpredictable bodily functions and irregular bowel movements. Bowel dysfunction hinders a stable life and introduces uncertainty, further intensifying the challenges they face. As they encounter various limitations in their daily lives, bowel problems become a central concern that significantly affects their overall QoL.

a) Challenges posed by unpredictable bodily functions

In the reviewed studies, patients with rectal cancer became increasingly aware of physical changes following surgery.^2^^,^^6^^,^^12^^,^^13^^,^^15^^,^^22^, ^23^, ^24^, ^25^, ^26^, ^27^, ^28^, ^29^, ^30^, ^31^ They experienced a loss of control over bowel movements,^2^^,^^12^^,^^15^^,^^22^, ^23^, ^24^, ^25^, ^26^, ^27^, ^28^, ^29^, ^30^, ^31^ leading to disruptions in their daily routines because of unpredictable patterns.^2^^,^^4^^,^^12^^,^^13^^,^^15^^,^^17^^,^^22^, ^23^, ^24^, ^25^, ^26^, ^27^, ^28^, ^29^^,^^31^ They endured persistent fatigue in their efforts to cope with unpredictable symptoms.^6^^,^^12^^,^^15^^,^^16^^,^^23^^,^^24^^,^^26^, ^27^, ^28^, ^29^, ^30^

*“And then I realize that I have to go to toilet again. I notice that this will happen very often today. But when it will happen, I do not know exactly in advance.**^22^**”*

*“I can go to the restroom 15–16 times within two hours at night. I get almost no sleep … For the first two months after surgery, I had to go even in the early morning. I got almost no sleep.**^30^**”*

b) Bowel movement at the core of daily life

Bowel movements become a central focus in the lives of patients with rectal cancer, owing to their unpredictable nature. Because of bowel problems, participants' activities were restricted by the availability of toilets,^4^^,^^6^^,^^10^^,^^12^^,^^15^^,^^17^^,^^22^^,^^26^, ^27^, ^28^^,^^30^, ^31^, ^32^, ^33^ and their food choices were constrained by the need for bowel control.^2^^,^^12^^,^^13^^,^^15^^,^^17^^,^^22^^,^^27^^,^^29^, ^30^, ^31^, ^32^ Additionally, their families had to adjust their daily lives to accommodate their bowel-related needs.^4^^,^^12^^,^^15^^,^^17^

*“I’ve gotten used to it, so I just make sure I’m somewhere near a toilet and have a clear path to run down. … So, when I’ve been out to eat, it has been good to know where it is, but it has gone well.**^25^**”*

*“If something special’s going on then it’s better not to eat. I daren’t eat in that case because it feels safer to just completely refrain.**^27^**”*

#### Theme Ⅱ. Living a life of isolation and disconnection

This theme consisted of two subthemes: social isolation and psychological distress from emotional instability. Patients with rectal cancer live isolated lives because of their physical and emotional limitations. They face social isolation and disconnection owing to increased physical limitations in social activities and various forms of psychological distress caused by bowel problems.

a) Social isolation

In the reviewed studies, participants with rectal cancer faced challenges in going out because of irregular bowel issues.^4^^,^^12^^,^^15^, ^16^, ^17^^,^^22^^,^^23^^,^^25^^,^^27^^,^^30^, ^31^, ^32^, ^33^ Their hobbies and leisure activities were often restricted by these issues.^4^^,^^12^^,^^15^^,^^17^^,^^23^^,^^27^^,^^30^^,^^31^^,^^33^ Physical limitations and difficulties in social interactions made it difficult for them to maintain intimate relationships,^4^^,^^12^^,^^15^^,^^23^^,^^25^^,^^30^^,^^31^^,^^33^ while social stigma further reduced their participation in social activities.^4^^,^^12^^,^^14^^,^^17^^,^^23^ Additionally, they struggled to sustain their work life.^15^, ^16^, ^17^^,^^25^

*“I had to go to the toilet 15 to 23 times, and at once! So I wasn't able to go out of the front door.**^33^**”*

*“I don’t think I can go play golf. … Even walks, I guess even, like, going for a walk after supper, you just have to be careful how far you go.**^25^**”*

b) Psychological distress from emotional instability

Participants in the reviewed studies felt frustrated due to bowel problems,^2^^,^^4^^,^^14^, ^15^, ^16^, ^17^^,^^22^^,^^26^, ^27^, ^28^, ^29^, ^30^, ^31^ fear of cancer recurrence^2^^,^^4^^,^^22^^,^^27^^,^^28^ and experienced guilt related to sexual difficulties.^2^^,^^4^^,^^15^^,^^16^^,^^25^^,^^31^ They were afraid that others would discover their condition^14^^,^^15^^,^^23^^,^^27^ and felt confused by some tips from health care providers or those around them.^2^^,^^12^, ^13^, ^14^^,^^25^^,^^27^, ^28^, ^29^^,^^33^

*“I felt urgency and then had incontinence! There were several times I accidently soiled my underwear, which made me upset and I wondered, ‘Why did it become so out of control?!’**^26^**”*

*“I am a very proud woman. It all has to be clean. And what could be dirtier than stool? It smells. I think shame is still deeply embedded in me. So in terms of intimacy, for example, that has also changed enormously.**^4^**”*

#### Theme Ⅲ. Finding a new balance in life

This theme contained three subthemes: psychological acceptance, developing personal strategies to regain control, and adapting to a changed daily life. Despite living with uncertainty and social disconnection, patients with rectal cancer strive to find a new sense of balance by psychologically accepting their condition, developing personal coping strategies, and adapting to their altered lives.

a) Psychological acceptance

Participants in the reviewed studies received emotional support from meaningful relationships^2^^,^^6^^,^^14^^,^^17^^,^^23^^,^^25^, ^26^, ^27^, ^28^^,^^31^, ^32^, ^33^, ^34^ and accepted their lives as they were.^4^^,^^14^^,^^17^^,^^22^^,^^29^ They felt grateful for the life they were able to live^6^^,^^15^^,^^17^^,^^23^^,^^25^, ^26^, ^27^^,^^29^ and sought comfort by comparing their situation with the suffering of others.^6^^,^^25^ Rather than suppressing or avoiding unpleasant experiences, participants wanted to pursue better psychological stability by naturally recognizing and accepting them.

*“Oh God, I’ve been through cancer surgery—what should I expect? I’ve been lucky! I think I’ve got a little handicap and I’ll have to live with that. … I’ve been given a second chance in life. And I’m going to take good care of it!**^27^**”*

*“You know what, nobody can see that I’m ill, and that is a good thing because no one is looking at me and talking about me. There’s a young girl in town, 19 years old, I think. She has lost a foot to cancer and everybody can see that.**^6^**”*

b) Developing personal strategies for regain control

The participants in the reviewed studies developed personal strategies to regain control over their bowel movements. They became more sensitive to their bodily signals^6^^,^^25^, ^26^, ^27^^,^^31^ and sought accurate symptom management information.^2^^,^^14^^,^^15^^,^^17^^,^^25^, ^26^, ^27^^,^^31^^,^^33^ They tried to manage the symptoms that they had difficulty controlling by managing their diet,^2^^,^^6^^,^^13^^,^^15^^,^^17^^,^^22^^,^^23^^,^^25^, ^26^, ^27^, ^28^, ^29^, ^30^^,^^32^^,^^34^ using medications such as loperamide, movicol, probiotics, and morphine to manage bowel function^2^^,^^6^^,^^15^^,^^23^^,^^25^^,^^27^, ^28^, ^29^, ^30^^,^^32^, and using pads to prevent incontinence.^2^^,^^17^^,^^22^^,^^23^^,^^25^^,^^28^^,^^29^^,^^32^

*“We had such a strong chilli stew here one day, and it was two days before I was normal again. So, I try to stay away from it.**^25^**”*

*“And I take 5 or 10 ​mg of morphine every night to get a good night’s sleep. I don’t take it to manage pain, it’s to calm my peristalsis.**^6^**”*

c) Adapting to a changed daily life

Participants in the reviewed studies adopted a new routine that differed from their pre-illness lifestyle. They established new bowel habits tailored to their patterns^6^^,^^15^^,^^17^^,^^22^^,^^25^, ^26^, ^27^, ^28^, ^29^, ^30^^,^^32^ and built trusting relationships with health care providers.^2^^,^^14^^,^^16^^,^^17^^,^^25^^,^^26^^,^^31^, ^32^, ^33^ Rather than hiding their illness, they made an effort to communicate openly^2^^,^^6^^,^^14^^,^^16^^,^^17^^,^^25^^,^^28^ and adjusted their lives by planning carefully before going out.^6^^,^^13^^,^^15^^,^^17^^,^^22^^,^^32^

*“I must finish going to the toilet in the morning, preferably 2–3 times. It’s rare that something happens before 11 a.m.**^25^**”*

*“I can rely on the care team, so I believe that ‘I can fight any problems.’**^26^**”*

*“I need to plan ahead if I’m going travelling … ehh … thenI’ll fast until I reach my destination, mostly because then I don’t need to make sure that a toilet is close by.**^6^**”*

### A conceptual model of the adaptation process among patients experiencing bowel dysfunction after low anterior resection for rectal cancer (Fig. 2)

This conceptual model illustrates the process of adaptation in patients with bowel dysfunction following LAR for rectal cancer. Patients initially experience unpredictable symptoms, which result in uncertainty in daily life. This uncertainty contributes to social isolation and psychological distress, creating a self-reinforcing cycle. Despite this cycle, patients strive to achieve balance in their lives. This adaptation process is facilitated by psychological acceptance, emotional support, and the development of personal strategies, which moderate the impact of psychological distress and support the transition toward stability.

**Fig. 2:**
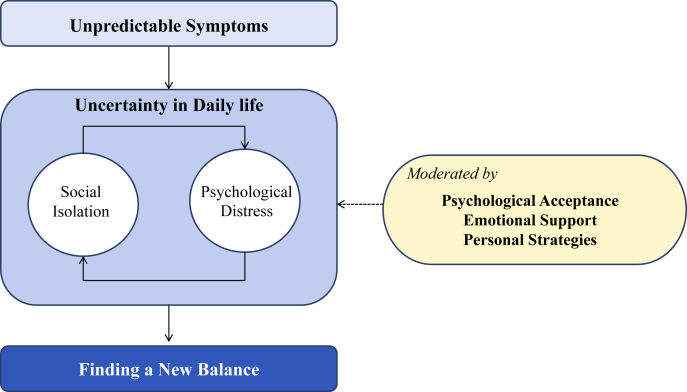
A conceptual model of the adaptation process among patients experiencing bowel dysfunction after low anterior resection for rectal cancer.

## Discussion

This meta-ethnographic synthesis identified three key themes reflecting patients' adaptation to bowel dysfunction after rectal cancer surgery. The first theme, “a life burdened by uncertainty,” highlighted the profound disruption to their daily routines due to unpredictable symptoms and a sense of loss of control. The second theme, “a life of isolation and disconnection,” captured their experience of social withdrawal and emotional distress because of embarrassment, shame, and/or fear of stigma. Finally, the third theme, “finding a new balance in life,” illustrated patients' proactive measures to regain stability through psychological acceptance, emotional support from relationships, and personalized coping strategies. Therefore, adaptation to LARS extends beyond symptom management; it involves the process of reconstructing one's identity, relationships, and sense of normalcy.

A significant finding of the present study is patients’ experience of uncertainty after undergoing LAR. Unpredictable symptoms disrupt daily life, making bowel movement a central concern. Their activities were limited to the toilet area, with food choices and family life centered on their bowel movements.

This theme corresponds closely to Mishel's Uncertainty in Illness Theory, which articulates uncertainty as the inability to determine the meaning of illness-related events owing to a lack of understanding of cues, ambiguity, or situational complexity.^35^^,^^36^ The patients in the present study experienced cognitive uncertainty as they struggled to interpret and manage unpredictable bowel symptoms in the absence of appropriate guidance or clear expectations. Such uncertainty induced a sense of loss of control, heightened psychological distress, and disrupted their daily routines. According to Mishel, adaptation is hampered when an individual appraises uncertainty as a threat rather than an opportunity, which was evident in our findings as the participants expressed fear, frustration, and reduced social engagement. Therefore, reducing uncertainty through patient-centered interventions and consistent communication from health care providers may be critical to fostering adaptive responses and improving patients' QoL.

The second significant finding of this study was that bowel dysfunction caused patients with rectal cancer who underwent LAR to live in isolation. Social isolation was shaped by both physical restrictions, such as constant bathroom awareness, and psychological distress associated with perceived stigma. Persistent anxiety further exacerbated social isolation, thereby limiting their daily functioning. Social isolation negatively impacts QoL and contributes to depressive and anxiety symptoms.^37^ Therefore, previous studies have suggested that participation in self-help groups can promote a sense of belonging and minimize isolation by allowing patients to share their experiences with others experiencing similar situations.^38^^,^^39^ Regular therapy with health care or mental health professionals may help alleviate emotional distress and support psychological stability.^40^ Additionally, bowel management programs that include dietary regulation and appropriate medication can enhance the predictability of bowel patterns, thereby boosting patients’ confidence in daily life.^41^ Furthermore, interventions such as meditation, psychotherapy, and stress management programs may reduce their fear and anxiety, thereby encouraging their engagement in social and personal activities.^42^^,^^43^

Nurses should utilize validated and condition-specific assessment tools to assess the impact of bowel dysfunction on patients’ psychosocial well-being. The Fecal Incontinence Quality of Life (FIQL) scale is widely used in colorectal cancer care to evaluate emotional distress, lifestyle limitations, and coping mechanisms associated with bowel dysfunction. Although FIQL does not fully capture the complexity of LARS—particularly fragmentation and clustering—it does offer valuable insight into the quality-of-life implications of fecal incontinence, which is a key component of LARS.^44^ Inclusion of the LARS Score, which specifically measures the severity of postoperative bowel dysfunction, enables more targeted assessment.^45^ Additionally, nurses can utilize brief psychosocial screening tools such as the Distress Thermometer to identify patients at risk of emotional distress and provide timely interventions.^46^

The third important finding of this study is that patients eventually achieved a sense of balance in their lives after surgery. They adapted to changes to regain psychological acceptance and control, supported by self-care, meaningful relationships, and health care providers. This process reflects the core elements of Taylor's Cognitive Adaptation Theory—meaning-making, mastery, and self-enhancement.^47^^,^^48^

A structured, phase-specific nursing intervention program based on the identified stages of adaptation may offer post-surgery support to patients. Survivorship navigation models can be customized to the LARS context by integrating pre-discharge education, early follow-up coaching, and long-term psychosocial adjustment counseling. A recent systematic review and meta-analysis emphasized that nurse-led interventions improved patients' QoL and alleviated psychological distress, such as anxiety and depression.^49^ These findings highlight the potential of nursing interventions—including bowel diary training, dietary adjustment education, and emotional support—to facilitate adaptation among patients experiencing LARS. By tailoring interventions to correspond with patients’ psychological stages, such as uncertainty, isolation, and reintegration, nurses can offer more personalized and effective care across the postoperative course.

Consistent with this theoretical perspective, patients described the establishment of a “new normal” despite physical and emotional challenges, ongoing uncertainty, and social disconnection. Unlike survivors of other cancers such as breast cancer, patients^50^^,^^51^ with rectal cancer experience unique struggles due to bowel-related symptoms that deeply impact their daily routines and social lives. These findings highlight the need for tailored interventions that address both physical symptoms and emotional adaptation to support successful lifestyle transitions. Health care providers should understand patients’ adaptation process to develop and provide various intervention strategies for enabling their adaptation through facilitators.

Considering the inclusion of studies from diverse cultural contexts, notable differences emerged between Eastern and Western participants in their experiences and coping strategies related to bowel dysfunction after rectal cancer surgery. Participants from Western countries (e.g., the UK, Netherlands, Sweden, and Switzerland) tended to describe their adaptation process in terms of autonomy, proactive self-management, and open communication with health care professionals.^2^^,^^22^^,^^23^^,^^27^, ^28^, ^29^^,^^32^^,^^33^ They stressed the significance of regaining control through practical adjustments, such as dietary modifications, routine planning, and seeking psychological support. The presence of multidisciplinary follow-up care—including nurses, stoma therapists, and psychologists—appeared to facilitate smoother transitions and improve adaptation.

In contrast, participants from Eastern settings (e.g., China, Taiwan, and Japan) often reported more passive or accepting coping mechanisms rooted in cultural values, such as self-restraint, deference to authority, and a desire to avoid shame or burdening others.^12^^,^^13^^,^^26^^,^^30^^,^^34^ Often, discussions around bowel-related symptoms were minimized or avoided, reflecting social taboos and fear of stigma. Family members contributed significantly in the adaptation process, both as sources of emotional support and as co-managers of dietary and lifestyle routines. However, limited access to specialized follow-up care and inadequate information were common barriers to successful adaptation.

Apart from cultural norms, patients’ adaptation trajectories may be influenced by differences in health care system structures. Participants in Western settings could access multidisciplinary support teams and structured follow-up care, which facilitated proactive coping. However, passive coping strategies and feelings of uncertainty among participants from Eastern contexts may be due to the limited continuity of care and lower availability of specialized services.

Digital health interventions can effectively complement traditional care by assisting symptom management and minimizing social isolation. A recent randomized controlled trial showed that a mobile health–based remote management intervention significantly improved QoL, bowel self-management behaviors, perceived social support, and symptom severity among patients with LARS over a six-month period.^52^ These findings suggest that digital tools—delivered through mobile platforms and supported by ongoing professional interaction—can assist patients in managing bowel dysfunction more confidently, particularly in case of limited in-person care or stigma.

Although the present review analyzed studies from various Asian countries, none of the included studies were conducted in South Korea, where sociocultural and dietary patterns present distinct features. In Korea, business-related social gatherings often include prolonged sitting, alcohol consumption, and intake of spicy or fermented foods, all of which may aggravate bowel symptoms or produce anxiety among individuals with LARS.^53^ Moreover, patients may not disclose their symptoms or seek professional support due to deeply ingrained social norms around embarrassment and saving face.^54^ These cultural nuances can impact their coping strategies and adaptation processes. Future research focusing on South Korean patients is required to address these culture-specific challenges and develop context-specific nursing interventions and educational strategies.

These findings have significant implications for nursing practice. Nurses should develop culturally sensitive and personalized care plans to support adaptation among rectal cancer survivors. These comprehensive plans should integrate psychosocial support, structured education, and clear communication strategies that respect patients’ values and cultural norms. Nurses have a significant role in providing individualized counseling, alleviating social isolation, and fostering psychological acceptance. In contexts with customary emotional restraint and stigma, family involvement and culturally appropriate educational materials may enhance patient support and engagement.

Although this meta-synthesis did not explore sex- or age-related differences, future studies should examine the impact of these differences in patients’ adaptation experiences, particularly in relation to psychological adjustment and social reintegration.

### Limitations

This study had several limitations. First, the findings have limited generalizability and transferability. Although the synthesis included 22 studies, differences were observed in participant characteristics, health care systems, and sociocultural contexts. Additionally, studies from low- and middle-income countries were scarce. Therefore, future research that includes more diverse and inclusive perspectives on adaptation to LARS is required. In addition, age-specific analyses were not feasible due to lack of disaggregated data in the original studies, which should be considered in future research. Nevertheless, the international scope of this review offers a comprehensive understanding of patients' experiences and can inform the development of globally implementable nursing interventions for bowel dysfunction. Second, although this review addressed existing limitations, such as limited database coverage and outdated literature, a qualitative meta-synthesis is inherently interpretative. Even though some degree of subjectivity is inevitable, the structured application of the meta-ethnographic method increases the credibility of the findings by ensuring a consistent theme interpretation across studies. Finally, the synthesis is shaped by researchers’ epistemological perspectives; meaning-making may still reflect subjective interpretation despite methodological rigor.

### Clinical implications

These findings have important implications for international nursing practice. The included studies encompassed diverse health care systems and cultural contexts and underscored the global nature of bowel dysfunction after rectal cancer surgery. Despite regional variations, the commonality of patients’ experiences suggests a pressing need for standardized evidence-based nursing approaches to manage bowel dysfunction across settings. Owing to the rising prevalence of colorectal cancer worldwide, nursing professionals must be equipped with culturally sensitive education and intervention strategies. Integrating this into nursing curricula and continuing education programs can enhance nurses' ability to provide person-centered care. Furthermore, international collaboration is required to develop tailored interventions and guidelines to support the adaptation and self-management of patients with LARS and ensure the provision of high-quality care.

In clinical settings, nurses can play a central role by offering anticipatory guidance on symptom changes, supporting individualized dietary and toileting plans, and facilitating timely referrals to specialists, such as stoma care nurses or mental health professionals. Within survivorship care, nurses are well-positioned to monitor psychosocial adjustment, lead patient and caregiver education, and advocate for resources that reflect patients’ cultural and personal values.

## Conclusions

This study provides insight into the experiences of patients with rectal cancer adapting to bowel dysfunction after LAR. It highlights challenges, such as uncertainty, social isolation, and psychological distress, and reveals patient-driven strategies for adaptation. These findings underscore the need for globally informed patient-centered nursing interventions that support holistic recovery beyond symptom management. Nurses play a critical role in delivering tailored self-care guidance, emotional support, and community resources. Embedding such care into structured rehabilitation and survivorship programs may improve patients’ QoL. These insights can also inform international nursing practice by contributing to culturally sensitive education and policy development to support cancer survivors.

Future research should explore culturally tailored intervention plans that reflect regional values and care systems, especially in underrepresented settings, such as South Korea. In-depth studies on the role of family in patients' adaptation as well as longitudinal research on patients’ evolving coping strategies are needed. These efforts may deepen our understanding of survivorship and contribute to more responsive and inclusive nursing practices.

## CRediT authorship contribution statement

**Eun Young KIM**: Data curation, Formal analysis, Funding acquisition, Investigation, Methodology, Software, Supervision, Validation, Visualization, Writing – original draft. **Min Jeong KIM**: Data curation, Investigation, Methodology, Software, Visualization, Writing – original draft. **Young Man KIM**: Conceptualization, Data curation, Formal analysis, Funding acquisition, Investigation, Methodology, Project administration, Resources, Software, Supervision, Validation, Visualization, Writing – original draft, Writing – review & editing. All authors have read and approved the final manuscript.

## Ethics statement

Not required.

## Data availability statement

The data that support the findings of this study are available on request from the corresponding author, YMK.

## Declaration of generative AI and AI-assisted technologies in the writing process

During the preparation of this work the authors used ChatGPT in order to assist with language translation. After using this tool/service, the authors reviewed and edited the content as needed and take full responsibility for the content of the publication.

## Funding

This work was supported by the National Research Foundation of Korea (NRF) grant (Grant No. RS-2024-00358888), and by another NRF grant (Grant No. 2021R1G1A1012978) funded by the ​Korea government Ministry of Science and ICT (MSIT). This work was supported by the Soonchunhyang University Research Fund. The funders had no role in considering the study design or in the collection, analysis, interpretation of data, writing of the report, or decision to submit the article for publication.

## Declaration of competing interest

The authors declare no conflict of interest.
