# Supplementary material for: Coping with bowel dysfunction after low anterior resection for rectal cancer: A qualitative synthesis
Source: Asia Pac J Oncol Nurs. 2025 Sep 18;12:100787. doi: 10.1016/j.apjon.2025.100787 (PMC12517071; doi:10.1016/j.apjon.2025.100787)
Supplement: Multimedia component 1 [file mmc1.docx]

**Supplementary Table S1.**

Search strategy

| Databases | Search No. | Query | Results |
| --- | --- | --- | --- |
| PubMed | #1 | “Colorectal Neoplasms”[MeSH] OR colorectal neoplasms[tiab] | 257,431 |
|  | #2 | “Colorectal Surgery”[MeSH] OR colorectal surgery[tiab] OR low anterior resection[tiab] OR ultra low anterior resection[tiab] OR ultra-low anterior resection[tiab] OR sphincter saving surgery[tiab] OR sphincter-saving surgery[tiab] OR sphincter preserving surgery[tiab] OR sphincter-preserving surgery[tiab] OR sphincter sparing surgery[tiab] OR sphincter-sparing surgery[tiab] | 16,881 |
|  | #3 | #1 AND #2 | 6,967 |
|  | #4 | Low Anterior Resection Syndrome[MeSH] OR “Defecation”[MeSH] OR “defecation*”[tiab] OR “Fecal Incontinence”[MeSH] OR “fecal incontinence”[tiab] OR “anterior resection syndrome”[tiab] OR “low anterior resection syndrome”[tiab] OR “bowel function”[tiab] OR “bowel dysfunction”[tiab] | 33,003 |
|  | #5 | “Interviews as Topic”[MeSH] OR “Interview”[Publication Type] OR interviews[tiab] OR “Anthropology, Cultural”[MeSH] OR “cultural anthropology”[tiab] OR ethnography[tiab] OR ethnographies[tiab] OR “Focus Groups”[MeSH] OR “focus group”[tiab] OR “focus groups”[tiab] OR “Grounded Theory”[MeSH] OR “grounded theory”[tiab] OR “Hermeneutics”[MeSH] OR hermeneutic[tiab] OR hermeneutics[tiab] OR “Personal Narrative”[Publication Type] OR “Narrative Medicine”[MeSH] OR narrative[tiab] OR narration[tiab] OR “Qualitative Research”[MeSH] OR “qualitative research”[tiab] OR experience[tiab] OR experiences[tiab] OR perspectives[tiab] OR perspective[tiab] OR views[tiab] OR phenomenology[tiab] | 2,208,618 |
|  | #6 | #3 AND #4 | 862 |
|  | #7 | #3 AND #5 | 869 |
|  | #8 | #3 AND #4 AND #5 | 140 |
| EMBASE | #1 | ‘colorectal cancer’/exp OR ‘colorectal cancer’:ab,ti OR ‘rectum cancer’/exp OR ‘cancer, rectum’:ab,ti OR ‘malignancy, rectum’:ab,ti OR ‘rectal cancer’:ab,ti OR ‘rectal malignancy’:ab,ti OR ‘rectum cancer’:ab,ti OR ‘rectum malignancy’:ab,ti OR ‘colorectal tumor’/exp OR ‘colorectal neoplasia’:ab,ti OR ‘colorectal neoplasm’:ab,ti OR ‘colorectal neoplasms’:ab,ti OR ‘colorectal tumor’:ab,ti OR ‘colorectal tumour’:ab,ti OR ‘tumor, colorectal’:ab,ti OR ‘tumour, colorectal’:ab,ti OR ‘rectum tumor’/exp OR ‘mass, rectum’:ab,ti OR ‘neoplasma recti’:ab,ti OR ‘pararectal tumor’:ab,ti OR ‘pararectal tumour’:ab,ti OR ‘rectal mass’:ab,ti OR ‘rectal neoplasm’:ab,ti OR ‘rectal neoplasms’:ab,ti OR ‘rectal tumor’:ab,ti OR ‘rectal tumour’:ab,ti OR ‘rectum mass’:ab,ti OR ‘rectum neoplasm’:ab,ti OR ‘rectum tumor’:ab,ti OR ‘rectum tumour’:ab,ti OR ‘retrorectal tumor’:ab,ti OR ‘retrorectal tumour’:ab,ti OR ‘tumor recti’:ab,ti OR ‘tumour recti’:ab,ti | 555,112 |
|  | #2 | ‘colorectal surgery’/exp OR ‘colon and rectal surgery (speciality)’:ab,ti OR ‘coloproctotomy’:ab,ti OR ‘colorectal surgery’:ab,ti OR ‘proctocolonic surgery’:ab,ti OR ‘surgery, colorectal’:ab,ti OR ‘low anterior resection’/exp OR ‘low anterior resection’:ab,ti OR ‘sphincter saving surgery’/exp OR ‘sphincter saving surgery’:ab,ti OR ‘sphincter preserving surgery’/exp OR ‘sphincter preserving surgery’:ab,ti OR ‘sphincter sparing surgery’/exp OR ‘sphincter sparing surgery’:ab,ti | 44,204 |
|  | #3 | #1 AND #2 | 20,786 |
|  | #4 | Low Anterior Resection Syndrome’/exp OR ‘defecation’/exp OR ‘bowel motion’:ab,ti OR ‘defaecation’:ab,ti OR ‘defecation’:ab,ti OR ‘feces incontinence’/exp OR ‘anal incontinence’:ab,ti OR ‘anus incontinence’:ab,ti OR ‘bowel incontinence’:ab,ti OR ‘encopresis’:ab,ti OR ‘faecal incontinence’:ab,ti OR ‘faeces incontinence’:ab,ti OR ‘fecal incontinence’:ab,ti OR ‘fecal incontinency’:ab,ti OR ‘feces incontinence’:ab,ti OR ‘incontinence, anal’:ab,ti OR ‘incontinentia alvi’:ab,ti OR ‘anterior resection syndrome’/exp OR ‘anterior resection syndrome’:ab,ti OR ‘low anterior resection syndrome’/exp OR ‘low anterior resection syndrome’:ab,ti OR ‘intestine function’/exp OR ‘intestine function’:ab,ti OR ‘bowel function’:ab,ti OR ‘function, intestine’:ab,ti OR ‘intestinal function’:ab,ti OR ‘bowel dysfunction’/exp OR ‘bowel dysfunction’:ab,ti | 704,715 |
|  | #5 | ‘qualitative research’/exp OR ‘qualitative research’:ab,ti OR ‘qualitative studies’:ab,ti OR ‘qualitative study’:ab,ti OR ‘interview’/exp OR ‘interview’ OR ‘interview guide’:ab,ti OR ‘interviews’:ab,ti OR ‘interviews as topic’:ab,ti OR ‘anthropology’/exp OR ‘anthropology’:ab,ti OR ‘focus group’/exp OR ‘focus group’:ab,ti OR ‘grounded theory’/exp OR ‘grounded theory’:ab,ti OR ‘hermeneutics’/exp OR ‘hermeneutics’:ab,ti OR ‘narrative’/exp OR ‘narrative’:ab,ti OR ‘experience’/exp OR ‘early experience’:ab,ti OR ‘experience’:ab,ti OR ‘perspective’/exp OR ‘perspective’:ab,ti | 2,646,718 |
|  | #6 | #3 AND #4 | 3,236 |
|  | #7 | #3 AND #5 | 2,491 |
|  | #8 | #3 AND #4 AND #5 | 473 |
| CINAHL | #1 | (MH “Colorectal Neoplasms+”) OR TI “colorectal neoplasms” OR AB “colorectal neoplasms” | 49,208 |
|  | #2 | TI “Colorectal Surgery” OR AB “Colorectal Surgery” OR TI “Colorectal cancer surgery” OR AB “Colorectal cancer surgery” OR TI “low anterior resection” OR AB “low anterior resection” OR TI “ultra low anterior resection” OR AB “ultra low anterior resection” OR TI “ultra-low anterior resection” OR AB “ultra-low anterior resection” OR TI “sphincter saving surgery” OR AB “sphincter saving surgery” OR TI “sphincter-saving surgery” OR AB “sphincter-saving surgery” OR TI “sphincter preserving surgery” OR AB “sphincter preserving surgery” OR TI “sphincter-preserving surgery” OR AB “sphincter-preserving surgery” OR TI “sphincter sparing surgery” OR AB “sphincter sparing surgery” OR TI “sphincter-sparing surgery” OR AB “sphincter-sparing surgery” | 2,735 |
|  | #3 | #1 AND #2 | 1,117 |
|  | #4 | MH “Low Anterior Resection Syndrome” OR MH “Defecation” OR (TI “defecation*”) OR (AB “defecation*”) OR MH “Bowel Function” OR (TI “bowel function”) OR (AB “bowel function”) OR MH “Fecal Incontinence” OR (TI “fecal incontinence”) OR (AB “fecal incontinence”) OR (TI “anterior resection syndrome”) OR (AB “anterior resection syndrome”) OR (TI “low anterior resection syndrome”) OR (AB “low anterior resection syndrome”) OR (TI “bowel dysfunction”) OR (AB “bowel dysfunction”) | 8,163 |
|  | #5 | MH “Interviews+” OR (TI “interview*”) OR (AB “interview*”) OR MH “Anthropology, Cultural” OR (TI “Anthropology, Cultural”) OR (AB “Anthropology, Cultural”) OR (TI “cultural anthropology”) OR (AB “cultural anthropology”) OR MH “Ethnographic Research” OR (TI “Ethnographic Research”) OR (AB “Ethnographic Research”) OR (TI “ethnograph*”) OR (AB “ethnograph*”) OR MH “Focus Groups” OR (TI “focus group*”) OR (AB “focus group*”) OR MH”Grounded Theory” OR (TI “grounded theory”) OR (AB “grounded theory”) OR MH “Phenomenology” OR (TI “phenomenology”) OR (AB “phenomenology”) OR (TI “hermeneutic*”) OR (AB “hermeneutic*”) OR MH “Narratives” OR MH “Narrative Medicine” OR (TI “narrative”) OR (AB “narrative”) OR (TI “narration”) OR (AB “narration”) OR MH “qualitative studies” OR (TI ‘‘qualitative stud*’’) OR (AB ‘‘qualitative stud*’’) OR (TI “experience*”) OR (AB “experience*”) OR (TI “perspective*”) OR (AB “perspective*”) OR (TI “view*”) OR (AB “view*”) | 1,139,250 |
|  | #6 | #3 AND #4 | 141 |
|  | #7 | #3 AND #5 | 216 |
|  | #8 | #3 AND #4 AND #5 | 46 |
| SCOPUS | #1 | TITLE-ABS-KEY(“colorectal cancer”) OR TITLE-ABS-KEY(“rectum cancer”) OR TITLE-ABS-KEY(“cancer, rectum”) OR TITLE-ABS-KEY(“malignancy, rectum”) OR TITLE-ABS-KEY(“rectal cancer”) OR TITLE-ABS-KEY(“rectal malignancy”) OR TITLE-ABS-KEY(“rectum cancer”) OR TITLE-ABS-KEY(“rectum malignancy”) OR TITLE-ABS-KEY(“colorectal tumor”) OR TITLE-ABS-KEY(“colorectal neoplas*”) OR TITLE-ABS-KEY(“colorectal tumor”) OR TITLE-ABS-KEY(“colorectal tumour”) OR TITLE-ABS-KEY(“tumor, colorectal”) OR TITLE-ABS-KEY(“tumour, colorectal”) OR TITLE-ABS-KEY(“rectum tumor”) OR TITLE-ABS-KEY(“mass, rectum”) OR TITLE-ABS-KEY(“neoplasma recti”) OR TITLE-ABS-KEY(“pararectal tumor”) OR TITLE-ABS-KEY(“pararectal tumour”) OR TITLE-ABS-KEY(“rectal mass”) OR TITLE-ABS-KEY(“rectal neoplasm”) OR TITLE-ABS-KEY(“rectal neoplasms”) OR TITLE-ABS-KEY(“rectal tumor”) OR TITLE-ABS-KEY(“rectal tumour”) OR TITLE-ABS-KEY(“rectum mass”) OR TITLE-ABS-KEY(“rectum neoplasm”) OR TITLE-ABS-KEY(“rectum tumor”) OR TITLE-ABS-KEY(“rectum tumour”) OR TITLE-ABS-KEY(“retrorectal tumor”) OR TITLE-ABS-KEY(“retrorectal tumour”) OR TITLE-ABS-KEY(“tumor recti”) OR TITLE-ABS-KEY(“tumour recti”) OR TITLE-ABS-KEY(“colon cancer”) OR TITLE-ABS-KEY(“colonic neoplasm*”) | 427,196 |
|  | #2 | TITLE-ABS-KEY(“colorectal surgery”) OR TITLE-ABS-KEY(“colon and rectal surgery”) OR TITLE-ABS-KEY(“coloproctotomy”) OR TITLE-ABS-KEY(“proctocolonic surgery”) OR TITLE-ABS-KEY(“surgery, colorectal”) OR TITLE-ABS-KEY(“low anterior resection”) OR TITLE-ABS-KEY(“ultra low anterior resection”) OR TITLE-ABS-KEY(“ultra-low anterior resection”) OR TITLE-ABS-KEY(“sphincter saving surgery”) OR TITLE-ABS-KEY(“sphincter-saving surgery”) OR TITLE-ABS-KEY(“sphincter-preserving surgery”) OR TITLE-ABS-KEY(“sphincter sparing surgery”) OR TITLE-ABS-KEY(“sphincter-sparing surgery”) | 26,934 |
|  | #3 | #1 AND #2 | 14,666 |
|  | #4 | TITLE-ABS-KEY(“defecation”) OR TITLE-ABS-KEY(“defaecation”) OR TITLE-ABS-KEY(“bowel motion”) OR TITLE-ABS-KEY(“feces incontinence”) OR TITLE-ABS-KEY(“anal incontinence”) OR TITLE-ABS-KEY(“anus incontinence”) OR TITLE-ABS-KEY(“bowel incontinence”) OR TITLE-ABS-KEY(“encopresis”) OR TITLE-ABS-KEY(“faecal incontinence”) OR TITLE-ABS-KEY(“faeces incontinence”) OR TITLE-ABS-KEY(“fecal incontinence”) OR TITLE-ABS-KEY(“fecal incontinency”) OR TITLE-ABS-KEY(“feces incontinence”) OR TITLE-ABS-KEY(“incontinence, anal”) OR TITLE-ABS-KEY(“incontinentia alvi”) OR TITLE-ABS-KEY(“anterior resection syndrome”) OR TITLE-ABS-KEY(“low anterior resection syndrome”) OR TITLE-ABS-KEY(“intestine function”) OR TITLE-ABS-KEY(“bowel function”) OR TITLE-ABS-KEY(“function, intestine”) OR TITLE-ABS-KEY(“intestinal function”) OR TITLE-ABS-KEY(“bowel dysfunction”) OR TITLE-ABS-KEY(“bowel dysfunction”) | 71,633 |
|  | #5 | TITLE-ABS-KEY(“qualitative research”) OR TITLE-ABS-KEY(“qualitative stud*”) OR TITLE-ABS-KEY(“interview*”) OR TITLE-ABS-KEY(“interview guide”) OR TITLE-ABS-KEY(“interviews as topic”) OR TITLE-ABS-KEY(“anthropology”) OR TITLE-ABS-KEY(“focus group”) OR TITLE-ABS-KEY(“grounded theory”) OR TITLE-ABS-KEY(“hermeneutics”) OR TITLE-ABS-KEY(“narrative”) OR TITLE-ABS-KEY(“narration”) OR TITLE-ABS-KEY(“experience*”) OR TITLE-ABS-KEY(“early experience*”) OR TITLE-ABS-KEY(“perspective*”) OR TITLE-ABS-KEY(“views”) OR TITLE-ABS-KEY(“phenomenology”) | 8,205,818 |
|  | #6 | #3 AND #4 | 1,625 |
|  | #7 | #3 AND #5 | 2,324 |
|  | #8 | #3 AND #4 AND #5 | 336 |
| Web of Science | #1 | TS=(“colorectal cancer”) OR TS=(“rectum cancer”) OR TS=(“cancer, rectum”) OR TS=(“malignancy, rectum”) OR TS=(“rectal cancer”) OR TS=(“rectal malignancy”) OR TS=(“rectum cancer”) OR TS=(“rectum malignancy”) OR TS=(“colorectal tumor”) OR TS=(“colorectal neoplasia”) OR TS=(“colorectal neoplasm”) OR TS=(“colorectal neoplasms”) OR TS=(“colorectal tumor”) OR TS=(“colorectal tumour”) OR TS=(“tumor, colorectal”) OR TS=(“tumour, colorectal”) OR TS=(“rectum tumor”) OR TS=(“mass, rectum”) OR TS=(“neoplasma recti”) OR TS=(“pararectal tumor”) OR TS=(“pararectal tumour”) OR TS=(“rectal mass”) OR TS=(“rectal neoplasm”) OR TS=(“rectal neoplasms”) OR TS=(“rectal tumor”) OR TS=(“rectal tumour”) OR TS=(“rectum mass”) OR TS=(“rectum neoplasm”) OR TS=(“rectum tumor”) OR TS=(“rectum tumour”) OR TS=(“retrorectal tumor”) OR TS=(“retrorectal tumour”) OR TS=(“tumor recti”) OR TS=(“tumour recti”) OR TS=(“colon cancer”) OR TS=(“colonic neoplasm*”) | 337,984 |
|  | #2 | TS=(“colorectal surgery”) OR TS=(“colon and rectal surgery”) OR TS=(“coloproctotomy”) OR TS=(“proctocolonic surgery”) OR TS=(“surgery, colorectal”) OR TS=(“low anterior resection”) OR TS=(“ultra low anterior resection”) OR TS=(“ultra-low anterior resection”) OR TS=(“sphincter saving surgery”) OR TS=(“sphincter-saving surgery”) OR TS=(“sphincter-preserving surgery”) OR TS=(“sphincter sparing surgery”) OR TS=(“sphincter-sparing surgery”) | 20,414 |
|  | #3 | #1 AND #2 | 8,117 |
|  | #4 | TS=(“defecation”) OR TS=(“defaecation”) OR TS=(“bowel motion”) OR TS=(“feces incontinence”) OR TS=(“anal incontinence”) OR TS=(“anus incontinence”) OR TS=(“bowel incontinence”) OR TS=(“encopresis”) OR TS=(“faecal incontinence”) OR TS=(“faeces incontinence”) OR TS=(“fecal incontinence”) OR TS=(“fecal incontinency”) OR TS=(“feces incontinence”) OR TS=(“incontinence, anal”) OR TS=(“incontinentia alvi”) OR TS=(“anterior resection syndrome”) OR TS=(“low anterior resection syndrome”) OR TS=(“intestine function”) OR TS=(“bowel function”) OR TS=(“function, intestine”) OR TS=(“intestinal function”) OR TS=(“bowel dysfunction”) OR TS=(“bowel dysfunction”) | 33,963 |
|  | #5 | TS=(“qualitative research”) OR TS=(“qualitative stud*”) OR TS=(“interview*”) OR TS=(“interview guide”) OR TS=(“interviews as topic”) OR TS=(“anthropology”) OR TS=(“focus group”) OR TS=(“grounded theory”) OR TS=(“hermeneutics”) OR TS=(“narrative”) OR TS=(“narration”) OR TS=(“experience*”) OR TS=(“early experience*”) OR TS=(“perspective*”) OR TS=(“views”) OR TS=(“phenomenology”) | 4,968,171 |
|  | #6 | #3 AND #4 | 1,115 |
|  | #7 | #3 AND #5 | 1,615 |
|  | #8 | #3 AND #4 AND #5 | 274 |
| Cochrane Library (CENTRAL) | #1 | MeSH descriptor: [Colorectal Neoplasms] explode all trees OR “colorectal neoplasm*”:ti,ab,kw | 15,463 |
|  | #2 | MeSH descriptor: [Colorectal Surgery] explode all trees OR (“colorectal surgery”):ti,ab,kw OR (“low anterior resection”):ti,ab,kw OR (“ultra low anterior resection”):ti,ab,kw OR (“ultra-low anterior resection”):ti,ab,kw OR (“sphincter saving surgery”):ti,ab,kw OR (“sphincter-saving surgery”):ti,ab,kw OR (“sphincter preserving surgery”):ti,ab,kw OR (“sphincter-preserving surgery”):ti,ab,kw OR (“sphincter sparing surgery”):ti,ab,kw OR (“sphincter-sparing surgery”):ti,ab,kw | 9,552 |
|  | #3 | #1 AND #2 | 4,001 |
|  | #4 | MeSH descriptor: [Low Anterior Resection Syndrome] OR MeSH descriptor: [Defecation] explode all trees OR (“defecation*”):ti,ab,kw OR MeSH descriptor: [Fecal Incontinence] explode all trees OR (“fecal incontinence”):ti,ab,kw OR (“anterior resection syndrome”):ti,ab,kw OR (“low anterior resection syndrome”):ti,ab,kw OR (“bowel function”):ti,ab,kw OR (“bowel dysfunction”):ti,ab,kw | 11,841 |
|  | #5 | MeSH descriptor: [Interviews as Topic] explode all trees OR (“Interview”):pt OR (“interview*”):ti,ab,kw OR MeSH descriptor: [Anthropology, Cultural] explode all trees OR (“cultural anthropology”):ti,ab,kw OR (“ethnography”):ti,ab,kw OR (“ethnographies”):ti,ab,kw OR MeSH descriptor: [Focus Groups] explode all trees OR (“focus group*”):ti,ab,kw OR MeSH descriptor: [Grounded Theory] explode all trees OR (“grounded theory”):ti,ab,kw OR MeSH descriptor: [Hermeneutics] explode all trees OR (“hermeneutic*”):ti,ab,kw OR (“Personal Narrative”):pt OR MeSH descriptor: [Narrative Medicine] explode all trees OR (“narrative”):ti,ab,kw OR (“narration”):ti,ab,kw OR MeSH descriptor: [Qualitative Research] explode all trees OR (“qualitative research”):ti,ab,kw OR (“experience*”):ti,ab,kw OR (“perspective*”):ti,ab,kw OR (“views”):ti,ab,kw OR (“phenomenology”):ti,ab,kw | 230,613 |
|  | #6 | #3 AND #4 | 339 |
|  | #7 | #3 AND #5 | 420 |
|  | #8 | #3 AND #4 AND #5 | 51 |
